# Influence of model complexity and problem formulation on the forces in the knee calculated using optimization methods

**DOI:** 10.1186/1475-925X-12-20

**Published:** 2013-03-07

**Authors:** Chih-Chung Hu, Tung-Wu Lu, Sheng-Chang Chen

**Affiliations:** 1 Institute of Biomedical Engineering, National Taiwan University, Taipei, Taiwan; 2 Department of Mechanical Engineering, Ming Chi University of Technology, Taipei, Taiwan; 3 Department of Orthopaedic Surgery, School of Medicine, National Taiwan University, Taipei, Taiwan

## Abstract

**Background:**

Predictions of the forces transmitted by the redundant force-bearing structures in the knee are often performed using optimization methods considering only moment equipollence as a result of simplified knee modeling without ligament contributions. The current study aimed to investigate the influence of model complexity (with or without ligaments), problem formulation (moment equipollence with or without force equipollence) and optimization criteria on the prediction of the forces transmitted by the force-bearing structures in the knee.

**Methods:**

Ten healthy young male adults walked in a gait laboratory while their kinematic and ground reaction forces were measured simultaneously. A validated 3D musculoskeletal model of the locomotor system with a knee model that included muscles, ligaments and articular surfaces was used to calculate the joint resultant forces and moments, and subsequently the forces transmitted in the considered force-bearing structures via optimization methods. Three problem formulations with eight optimization criteria were evaluated.

**Results:**

Among the three problem formulations, simultaneous consideration of moment and force equipollence for the knee model with ligaments and articular contacts predicted contact forces (first peak: 3.3-3.5 BW; second peak: 3.2-4.2 BW; swing: 0.3 BW) that were closest to previously reported theoretical values (2.0-4.0 BW) and *in vivo* data telemetered from older adults with total knee replacements (about 2.8 BW during stance; 0.5 BW during swing). Simultaneous consideration of moment and force equipollence also predicted more physiological ligament forces (< 1.0 BW), which appeared to be independent of the objective functions used. Without considering force equipollence, the calculated contact forces varied from 1.0 to 4.5 BW and were as large as 2.5 BW during swing phase; the calculated ACL forces ranged from 1 BW to 3.7 BW, and those of the PCL from 3 BW to 7 BW.

**Conclusions:**

Model complexity and problem formulation affect the prediction of the forces transmitted by the force-bearing structures at the knee during normal level walking. Inclusion of the ligaments in a knee model enables the simultaneous consideration of equations of force and moment equipollence, which is required for accurately estimating the contact and ligament forces, and is more critical than the adopted optimization criteria.

## Introduction

The knee joint plays a pivotal role in the normal function of the lower extremity during activities of daily living. The function of the knee relies upon a well-coordinated mechanical interaction between its force-bearing structures, including muscles, ligaments, menisci, articular cartilage and posterior capsule [1,2]. Knowledge of the forces transmitted by these force-bearing structures is essential for understanding and evaluating the function of the joint (normal or pathological), as well as for relevant clinical applications such as design and implantation of joint replacements. However, direct measurement of these forces *in vivo* is only possible in exceptional conditions such as through instrumented prostheses [3-6]. Therefore, mathematical modeling in conjunction with non-invasive experimental measurements has been – and still remains – the most commonly used approach for predicting the forces transmitted by the various force-bearing structures in the musculoskeletal system [7-12].

Basically, the process of estimating the forces transmitted by the force-bearing structures of the knee joint using a mathematical modeling approach involves two stages. The first stage is the calculation of the resultant forces and moments at the joint center relative to its distal segment (i.e., tibia) through the inverse dynamics analysis of measured kinematic and kinetic data [13,14]. The second stage is the geometrical modeling of the musculoskeletal system of the joint, giving the lines of action and lever arm vectors of each of the modeled force-bearing structures including the ligaments, muscles and articular surfaces [11,15]. Since the resultant forces and moments have to be provided by the force-bearing structures, the two force systems, namely the system of the resultant forces and moments, and the system of all the force-bearing structures, are equipollent. Two force systems are equipollent (equally powerful) if they have the same total force and total moment about the same point, e.g., knee joint center. This is different from the definition of equilibrium that requires that the sums of all external forces and moments be zero. When using the equations of equipollence, the articular surfaces are often modeled as rigid and the ligaments as non-extensible (e.g. [7]). At each time instance during the movement, the equations of force and moment equipollence at the joint center can then be used to distribute the resultant forces and moments to the individual force-bearing structures.

Given the resultant forces and moments via inverse dynamics analysis, and the lines of action and lever arm vectors of the force-bearing structures determined by the geometrical model, the only unknowns left to be determined are the magnitudes of the forces transmitted by the individual force-bearing structures. However, owing to the high degree of mechanical redundancy of the musculoskeletal system, solving the resulting simultaneous equations of force and moment equipollence remains a great challenge. For example, in the locomotor system there are at least 47 muscles in each leg influencing locomotion [16]. The equations available for dynamic equilibrium, however, are far too few to determine uniquely all the forces in the muscles, as well as other force-bearing structures simultaneously. In other words, there are an infinite number of solutions to this problem. One way to resolve this problem is to reduce the number of unknowns based on mechanical and physiological considerations to make the problem determinate such as combining muscles of similar function as a group [17,18]. Another more widely used approach is to select a unique solution from the infinite number of solutions based on certain optimization principles such as minimization of the sum of muscle forces [8-10,19-22].

Previous studies using optimization approaches have focused mainly on exploring the optimization principles (objective functions) that best predicted muscle recruitment patterns. The constraints considered have thus received less attention and are largely simplified. Even though theoretically both force and moment equipollence should be considered, as was done in some of these studies [9,10,23], most studies considered only equations of moment equipollence [8,16,19,20,24]. It remains unclear whether further inclusion of force equipollence equations to the force-distribution problem would affect the optimum solution given the same objective function.

Another concern about the consequences of the exclusion of the force equipollence is the subsequent calculation of the forces in the passive structures. In previous studies, the ligaments restraining the joint were either not modeled in detail [16] or were simply ignored [23]. Therefore, some studies calculated the muscle and contact forces first, considering only moment equipollence in their optimization problems. With the predicted muscle and contact forces the equations of force equipollence were then used to calculate the unbalanced forces, shear components of which were subsequently attributed to the restraining structures, i.e., the ligaments [10]. Whether the unbalanced shear forces are indeed equal to the shear component of the resultant ligament forces requires further clarification. In other studies that considered only moment equipollence as a result of modeling without ligaments, only muscle and/or contact forces were predicted assuming that ligaments contributed little to the joint resultant moments [20]. No attempt was made to use the equations of force equipollence to calculate the unbalanced forces or restraining forces, or to check whether the predicted forces of muscles and/or contact surfaces satisfied the force equipollence equations.

In the literature, various objective functions have been proposed to predict muscle recruitment patterns and force magnitudes for various joints using models of different complexities and different problem formulations [8,23-25]. Seireg and Arvikar [23] compared 14 criteria for predicting muscles forces in the lower limb during static postures and found that there was more than one criterion that could reasonably predict muscle activity patterns according to surface electromyography (EMG). Collins used a 2D model of the lower limb to evaluate the ability of various optimization criteria in predicting muscle activity patterns during level walking and found that, of the six tested criteria, five predicted similar patterns of muscle activity over a gait cycle [25]. In contrast, in the studies by Herzog and Leonard [24] and Challis and Kerwin [8], all the tested criteria failed to predict muscle force patterns that were consistent with the experimental data.

Instead of using EMG data for the validation of the predicted muscle forces and optimization criteria, EMG-assisted optimization methods take EMG data as input and estimate individual muscle forces following their EMG profiles by minimizing a given objective function subject to moment equipollence constraints [26]. With the same optimization criterion and moment constraints these methods were found to predict more physiological muscle force patterns, such as antagonist co-contractions, agonist synergy or other forms of muscle force patterns, than optimization methods without EMG were able to [26-28]. However, similar to optimization methods, force equipollence constraints were not included in previous EMG-assisted optimization formulations. Another limitation with EMG-assisted optimization is that muscles without EMG data, such as deep muscles, may not be accurately represented in the formulation.

The above literature review indicates that further study is required to answer the question whether the level of model complexity (i.e., with or without ligaments) and problem formulation (i.e., moment equipollence with or without force equipollence) will affect the ability of optimization-based methods in predicting forces transmitted by the force-bearing structures of a joint, including muscles, ligaments and articular contact surfaces. Therefore, the main purpose of this study was to investigate the influence of model complexity (with or without ligaments), problem formulation (moment equipollence with or without force equipollence) and optimization criteria on the prediction of the forces transmitted by the force-bearing structures in the knee using a three-dimensional musculoskeletal model of the locomotor system with a knee model that included all the major force-bearing structures, including muscles, ligaments and contact surfaces.

## Methods

Ten healthy male adults (age: 23.2 ± 2.1 years; height: 161.1 ± 8.1 cm, mass: 56.4 ± 8.6 kg) participated in this study with written informed consent. They were free of any history of neuromusculoskeletal diseases or impairments. Approval for this study was provided by the Institutional Ethics Review Committee. For each subject, a total of 28 infrared retroreflective markers were attached to specific bony landmarks on each limb for the description of the motion of the segments, including anterior superior iliac spines (ASIS), posterior superior iliac spines (PSIS), greater trochanter, mid-thigh, medial and lateral femoral epicondyles, tibial tuberosity, head of fibula, medial and lateral malleoli, calcaneus, navicular tuberosity and the base of the fifth metatarsal [29]. Subjects were asked to walk along an 8-m level walkway in a gait laboratory while the three-dimensional (3D) trajectories of the markers were measured with a 7-camera motion analysis system (VICON 512, Oxford Metrics, U.K.) at a sampling rate of 120 Hz. The ground reaction forces (GRF) were measured synchronously with two force platforms (AMTI, Mass., U.S.A.) at a sampling rate of 600 Hz. A second-order Butterworth low-pass filter with a cut-off frequency of 15 Hz was used to filter both the kinematic and force plate data [30]. Motion data from at least three successful trials for each subject were collected for subsequent analysis.

A validated 3D model of the human locomotor system [11] was adopted for the analysis of the measured motion data. The human pelvis-leg apparatus was modeled as four rigid body segments, namely the pelvis, thigh, shank and foot, connected by model joints. The hip was modeled as a ball-and-socket joint and the ankle as a two-hinge complex. The mobility of the tibiofemoral joint was controlled by a parallel spatial mechanism, formed by the isometric fibers of the anterior cruciate ligament (ACL), posterior cruciate ligament (PCL) and medial collateral ligament (MCL) as inextensible elements, as well as the lines defining the contact normals of the medial and lateral tibial plateau [31]. As a first approximation, the tibial condyles were modeled as planar rigid surfaces, the femoral condyles as spherical surfaces, and the tibia and femur were modeled as rigid bodies which maintained contact continuously in both compartments. These conditions specified a single point of tibiofemoral contact in each compartment. Formulae and model parameters describing the geometry of the knee model were taken from Wilson *et al.*[31].

As shown by Wilson *et al.*, the parallel spatial mechanism model of the knee has one degree of mobility so prescription of one model variable (e.g., flexion angle) is enough to determine uniquely the motion of the model knee. Following Wilson *et al.*, the flexion angle was taken as the variable describing the degree of mobility of the knee in the current study. Therefore, given the knee flexion angle, the motion of the model bones and all the force-bearing structures involved in the model are determined, including the lines of action of the ligaments and the contact normals.

The knee extensor mechanism, composed of the patellofemoral joint, patellar and quadriceps tendons, was also included in the model. The patella was represented as a point, namely the intersection of the quadriceps and patellar tendons [15,32,33]. In conjunction with the parallel spatial mechanism model, the knee extensor mechanism model takes into account the rolling motion of the femur on the tibia, as well as the so-called screw home motion [34,35], determining the change of geometry of the patellar tendon, quadriceps tendon and patellofemoral articular contact normal throughout the full range of knee flexion. Thirty-four muscles or muscle groups were included, represented by single lines joining their origins and insertions, wrapping around the underlying bones when necessary. Note that the ligaments and muscles were modeled as pure force generators. Therefore, they were fully described by their origins and insertions, as well as by the wrappings around the bones, giving their lines of action and lever arm lengths (moment arms).

The model was customized to individual subjects using homogeneous scaling techniques suggested by Brand *et al.*[36] and White *et al.*[37]. The homogeneous scaling matrices for each of the body segments were determined by minimizing the overall differences between the measured and model-determined marker coordinates on the segment in a least squares sense. These marker coordinates included those of the skin markers, as well as the joint centers as virtual markers. The center of rotation of the hip was estimated using a functional method [38]. For the knee and the ankle models, markers around the joints, including those on the femoral epicondyles and the malleoli, were used for scaling purposes.

The measured skin marker trajectories and force plate data were entered into the model to calculate the intersegmental resultant forces and moments at the joint centers during gait using inverse dynamics analysis. Inertial properties of the segments were determined using Dempster’s coefficients [39]. Skin marker movement artefacts were minimized using the Global Optimization Method (GOM) [40]. The GOM is based on the search for an optimal pose of the multi-link model of the locomotor system for each data frame such that the overall differences between the measured and model-determined marker coordinates are minimized in a weighted least squares sense, throughout all the body segments, while subject to the model joint constraints. Segments were assigned different weightings using their residual errors as a guide. The GOM considers measurement error distributions in the system and provides an error compensation mechanism between body segments in order to reduce skin movement artefacts. More details of the mathematical descriptions of the method and the determination of the weightings can be found in Lu and O’Connor [40].

For the present study, only the mechanics of the knee were considered so the resultant force

$$\vec{R}$$

and moment

$$\vec{M}$$

at the knee joint center, defined as the midpoint of the inter-condylar line, were transformed to the body-embedded coordinate system of the shank segment with the origin located at the tibial tuberosity, the x-axis directed anteriorly, y-axis superiorly and z-axis to the right. These resultant forces and moments were then distributed to the individual force-bearing structures considering force and moment equipollence as follows:

(1) $$\sum_{i=1}^{12}F_{i}^{m}{\vec{l}}_{i}^{m}+\sum_{j=1}^{4}F_{j}^{l}{\vec{l}}_{j}^{l}+\sum_{k=1}^{2}F_{k}^{c}{\vec{l}}_{k}^{c}=\vec{R}$$

(2) $$\sum_{i=1}^{12}{\vec{d}}_{i}^{m}\times \left(F_{i}^{m}{\vec{l}}_{i}^{m}\right)+\sum_{j=1}^{4}{\vec{d}}_{j}^{l}\times \left(F_{j}^{l}{\vec{l}}_{j}^{l}\right)+\sum_{k=1}^{2}{\vec{d}}_{k}^{c}\times \left(F_{k}^{c}{\vec{l}}_{k}^{c}\right)=\vec{M}$$

where $F_{i}^{m},\;F_{j}^{l}\;\text{and}\;F_{k}^{c}$ are force magnitudes of the muscles, ligaments and articular surfaces, respectively;

$${\vec{l}}_{i}^{m}$$,

$${\vec{l}}_{j}^{l}$$

and

$${\vec{l}}_{k}^{c}$$

are the corresponding unit vectors defining their lines of action; and

$${\vec{d}}_{i}^{m}$$,

$${\vec{d}}_{j}^{l}$$

and

$${\vec{d}}_{k}^{c}$$

are the corresponding lever-arm vectors pointing from the joint center to the insertions at the shank segment. Twelve muscles affecting the mechanics of the knee were considered, namely rectus femoris, semitendinosus, semimembranosus, biceps femoris (long and short heads), gastrocnemius (medial and lateral heads), vastus intermedius, vastus medialis, vastus lateralis, as well as the gluteus maximus and tensor fasciae latae that span the knee through the iliotibial tract. The ligaments included in the Wilson knee model [31], namely the ACL, PCL, MCL, the lateral collateral ligament (LCL), and the medial and lateral contact forces were also considered. Therefore, there were six equipollence equations in terms of 18 unknowns to be solved.

The most essential consideration when applying optimization techniques is the problem formulation, including the choice of design variables, the definition of a feasible region and the selection of a proper objective function (criterion). In the current study, force magnitudes of the muscles, ligaments and contact forces were chosen as design variables. Equations of mechanical equipollence at the joint center were the main equality constraints which formed the feasible region. Considering different design variable sets and equipollence equations at the knee, three types of problem formulation were constructed. The first, denoted RM, considered muscle and articular contact force magnitudes as design variables, and moment equipollence as the constraint. The second, denoted RML, considered muscle, ligament and articular contact force magnitudes as design variables, and only moment equipollence as the constraint. The third, denoted RFML, considered muscle, ligament and articular contact force magnitudes as design variables, and both the force and moment equipollence as constraints. Each type of formulation included eight objective functions commonly used in the literature, namely sum of muscle forces

$$\left(J1=\sum_{i=1}^{12}F_{i}^{m}\right)$$,

sum of quadratic muscle forces

$$\left(J2=\sum_{i=1}^{12}{\left(F_{i}^{m}\right)}^{2}\right)$$,

sum of cubic muscle forces

$$\left(J3=\sum_{i=1}^{12}{\left(F_{i}^{m}\right)}^{3}\right)$$,

maximum of muscle forces

$$\left(J4=\max_{i=1,12}\left(F_{i}^{m}\right)\right)$$,

sum of muscle stresses

$$\left(J5=\sum_{i=1}^{12}\frac{F_{i}^{m}}{A_{i}}\right)$$,

sum of quadratic muscle stresses

$$\left(J6=\sum_{i=1}^{12}{\left(\frac{F_{i}^{m}}{A_{i}}\right)}^{2}\right)$$,

sum of cubic muscle stresses

$$\left(J7=\sum_{i=1}^{12}{\left(\frac{F_{i}^{m}}{A_{i}}\right)}^{3}\right)$$,

and maximum of muscle stresses

$$\left(J8=\max_{i=1,12}\left(\frac{F_{i}^{m}}{A_{i}}\right)\right)$$.

The first four were force-based objective functions and the last four were stress-based ones.

The stress of a muscle is defined as the transmitted force (*F*_*i*_^*m*^) divided by its physiological cross-sectional area (PCSA or *A*_*i*_). The PCSA data for the muscles were obtained from Wickiewicz *et al.*[41] and Winter [39]. A Quasi-Newton procedure, Sequential Quadratic Programming (SQP) [42], was used to solve the resulting constrained optimization problems. In the solution process, a large upper bound of 5000 N was set for the ligament and muscle forces, which enabled us to check whether different formulations with different objective functions would be able to predict reasonable or physiological forces in the muscles and ligaments.

Since RM and RML considered only moment equipollence constraints, the forces calculated using these two formulations might not satisfy force equipollence equations. In the current study, the unbalanced force vector

$$\vec{e}$$,

defined as

(3) $$\vec{e}=\sum_{i=1}^{12}F_{i}^{m}{\vec{l}}_{i}^{m}+\sum_{k=1}^{2}F_{k}^{c}{\vec{l}}_{k}^{c}-\vec{R},$$

for RM, and

(4) $$\vec{e}=\sum_{i=1}^{12}F_{i}^{m}{\vec{l}}_{i}^{m}+\sum_{j=1}^{4}F_{j}^{l}{\vec{l}}_{j}^{l}+\sum_{k=1}^{2}F_{k}^{c}{\vec{l}}_{k}^{c}-\vec{R},$$

for RML, provided a measure of the error associated with the sole use of moment equipollence for force predictions.

RML and RFML considered the magnitude of the ligament forces so the total calculated forces of the knee ligament forces

$$\vec{L}$$,

defined as

(5) $$\vec{L}=\sum_{j=1}^{4}F_{j}^{l}{\vec{l}}_{j}^{l},$$

were obtained for both RML and RFML, and their differences were then calculated as an index to evaluate the performance of the RML.

## Results

**Figure 1:**
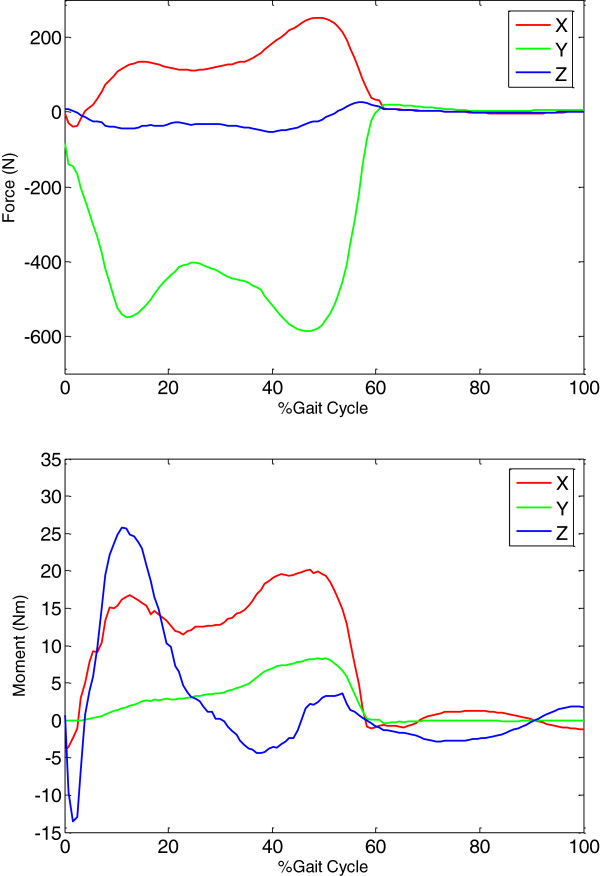
**Knee resultant forces and moments during gait.** Resultant forces $\vec{R}$ and moments $\vec{M}$ at the knee joint in a typical subject during level walking. For the resultant forces, X is the anterior (+)/posterior (−) component; Y is the superior (+)/inferior (−) component; and Z is the lateral (+)/medial (−) component. For the resultant moments, X is the adductor (+)/abductor (−) component; Y is the internal (+)/external (−) rotator component; and Z is the extensor (+)/flexor (−) component.

**Figure 2:**
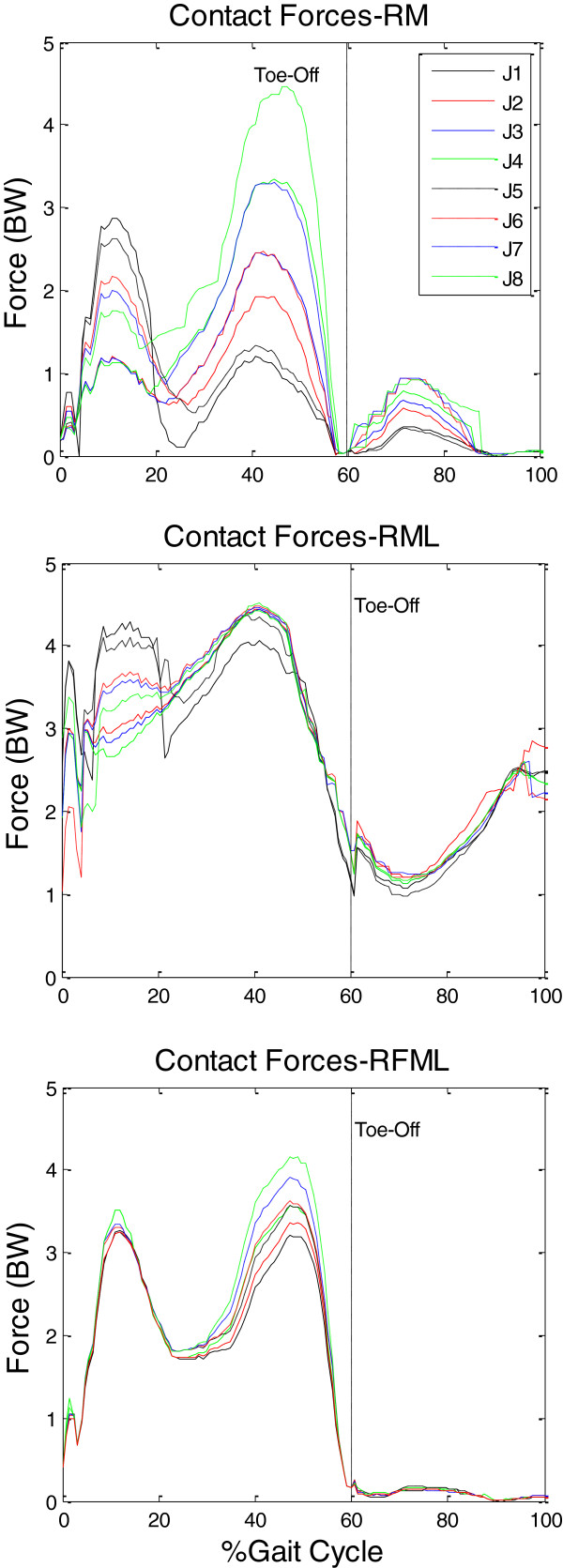
**Joint contact forces.** Joint contact forces calculated using three problem formulations (RM, RML and RFML) with eight objective functions (J1-J8).

With the resultant forces

$$\vec{R}$$

and moments

$$\vec{M}$$

at the knee joint calculated using inverse dynamics analysis (Figure 1), the forces transmitted in the muscles, articular surfaces and ligaments were calculated using the three formulations with different objective functions. The joint contact forces calculated using the three formulations (i.e., RM, RML and RFML) were different both in patterns and magnitudes (Figure 2). For different objective functions, the RM formulation predicted different results, the first peak of the contact force varying from 1.0 to 2.8 BW and the second peak varying between 1.0 and 4.5 BW. Differences in the calculated contact forces for different objective functions were also found in the RML formulation, the peak values during stance ranging from 3 to 4.5 BW and being as large as 2.5 BW during swing phase (Figure 2). These variations were largely reduced for the RMFL formulation, for which the first peak of the contact force ranged from 3.3 to 3.5 BW and the second peak ranged from 3.2 to 4.2 BW.

**Figure 3:**
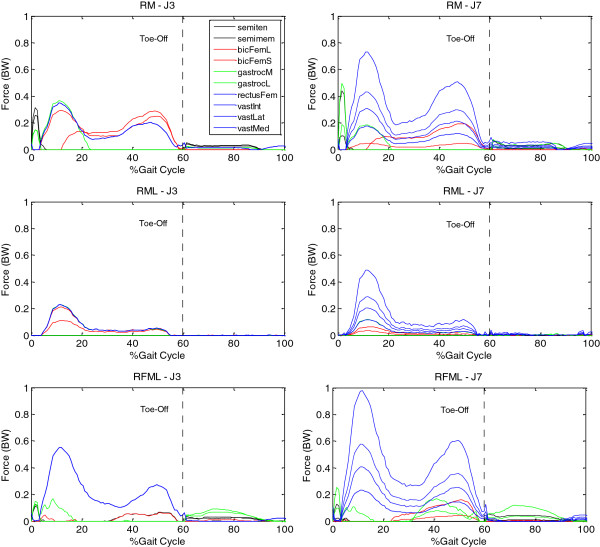
**Muscle recruitment and force patterns using J3 and J7.** Muscle recruitment and force patterns using RM, RML and RFML formulations with a typical force-based objective (J3) and a typical stress-based objective (J7).

Muscle recruitment patterns appeared to be quite different for different formulations, and between force-based and stress-based objective functions, as can be seen in the results of a typical force-based objective (J3) and a stress-based objective (J7) (Figure 3). Given the same formulation, muscle recruitment patterns for other muscle force-based objective functions (J1, J2 and J4) were similar to those of J3, while those for other muscle stress-based objectives (J5, J6 and J8) were similar to those of J7. Overall, the muscle stress-based objectives tended to predict more active muscles than the muscle force-based objectives (Figure 3).

**Figure 4:**
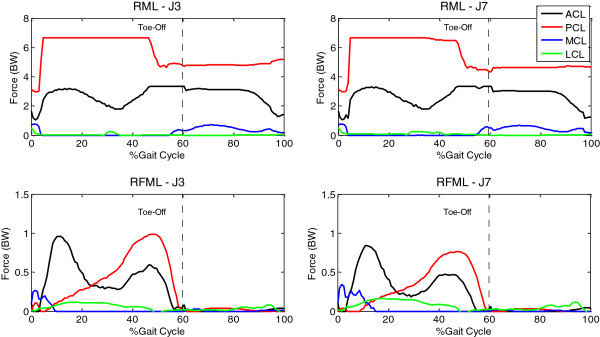
**Ligament forces using J3 and J7.** Ligament forces calculated using RML and RFML with a typical force-based objective (J3) and a typical stress-based objective (J7).

While the ligament forces could not be calculated using the RM formulation, the ligament forces calculated using RML and RFML were very different, both in pattern and magnitude, but the differences between objective functions with the same formulation were small (Figure 4). The forces of the ACL and PCL calculated using RML were several times body weight, while those using RFML were less than the body weight. With RML, the calculated ACL forces ranged from 1 BW to 3.7 BW, and those of the PCL from 3 BW to 7 BW. The RML also predicted MCL and LCL forces during the swing phase. With the additional inclusion of the force equipollence, the RFML predicted ligament forces that were all less than 1.0 BW, and appeared to be independent of the objective functions used (Figure 4). In contrast to RML, the RFML predicted MCL and LCL forces during the stance phase.

**Figure 5:**
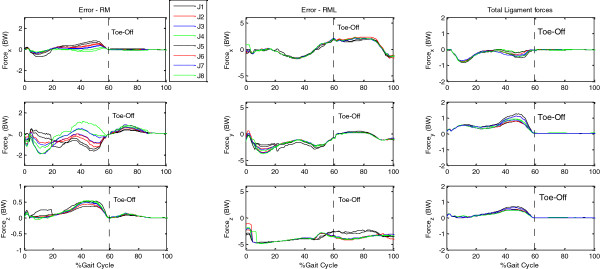
**Unbalanced force vectors and resultant ligament forces.** Unbalanced force vectors for both RM and RML with the eight objective functions (J1-J8). The resultant ligament forces $\vec{L}$ for RFML are also shown. X is the anterior (+)/posterior (−) component; Y is the superior (+)/inferior (−) component; and Z is the lateral (+)/medial (−) component.

Large unbalanced forces were found for both RM and RML (Figure 5). Considering only moment equipollence and without modeling the ligaments, the RM showed a maximum unbalanced force of about two times body weight (Figure 5) with a maximum RMS value of about 1.3 BW over the gait cycle (Table 1). With RML, apart from the muscle forces, greater ligament forces were required to meet the moment equipollence because the lever arm lengths of the ligaments were much smaller than those of the muscles. These overestimated ligament forces and the muscle forces did not satisfy the force equipollence, leaving a maximum unbalanced force of about −4.4 BW (Figure 5). Compared to the ligament forces calculated by the RFML, a maximum RMS difference of about 7 BW over the gait cycle was found for the RML (Table 1).

**Table 1:** Unbalanced forces and ligament forces

**RMS values**		**J1**	**J2**	**J3**	**J4**	**J5**	**J6**	**J7**	**J8**
Unbalanced force vector for RM	x	0.43	0.37	0.32	0.23	0.39	0.30	0.25	0.19
	y	1.15	1.21	1.12	0.94	1.03	0.86	0.74	0.71
	z	0.09	0.06	0.07	0.06	0.08	0.06	0.05	0.06
Unbalanced force vector for RML	x	1.23	1.42	1.39	1.40	1.21	1.39	1.26	1.40
	y	1.70	1.76	1.77	1.79	1.72	1.74	1.74	1.75
	z	3.59	3.88	3.84	3.84	3.62	3.76	3.77	3.80
Differences between ligament force using RML and RFML	x	2.39	3.06	2.92	2.93	2.41	2.91	2.85	2.92
	y	4.47	4.71	4.66	4.69	4.56	4.66	4.65	4.64
	z	3.26	3.30	3.28	3.29	3.32	3.21	3.24	3.20

Root-mean-squared (RMS) values of the unbalanced force vector components using RM and RML formulations, as well as RMS differences between the ligament force components using RML and RFML formulations. (Unit: BW).

## Discussion

The current study aimed to investigate the influence of model complexity, i.e., with or without considering ligaments, and problem formulations on the performance of various optimization criteria in predicting forces transmitted by the force-bearing structures at the knee, namely muscles, ligaments and articular contacts, during normal level walking. Three different problem formulations in combination with eight objective functions were considered. The results supported the hypotheses that, compared to other formulations, simultaneous consideration of force and moment equipollence produced more reasonable force estimations that were also less affected by the optimization criteria employed.

Among the three problem formulations, the RMFL predicted knee contact forces that were in good agreement with results reported in previous studies, both in patterns and magnitudes [5,18,43]. The results did not appear to be affected by the objective functions used. In contrast, the knee contact forces calculated using the other two formulations were quite different between objective functions (Figure 2). With the RM formulation, the first peak of the contact force occurred at around 10% gait cycle (GC) with magnitudes varying from 1.0 to 2.8 BW, and the force magnitudes of the second peak at around 40% GC varied between 1.0 and 4.5 BW. These force values for both peaks and the occurrence of the second peak were quite different from the *in vivo* data reported in the literature which showed a force range of 2–3 BW, and a second peak at about 50% GC [5,43]. During swing phase the contact forces calculated with RM rose to a maximum of 1.0 BW, which were also too large compared to those previously measured *in vivo*[5,43]. The great variability in the overestimated joint contact forces using RM appeared to be the result of the different muscle recruitment patterns predicted using different objective functions because a large proportion of joint contact forces come from muscles [6,18,40]. This dependence between the muscle recruitment patterns, and thus the magnitude of the joint contact force, and objective functions seemed to be related to the simplification of the knee model used. Previous studies without taking ligament effects into consideration have failed to produce a muscle recruitment pattern that matches the measured EMG well [11].

Even though ligaments were considered in the RML formulation, and the variability of contact force magnitudes was reduced, the calculated contact forces were still different between objective functions (Figure 2). The patterns of the calculated contact forces were quite different from the two-peak patterns observed in previous experimental studies [5]. The curves fluctuated throughout the gait cycle, with the peaks ranging from 3 to 4.5 BW. The contact forces increased to as large as 2.5 BW during swing phase, which disagrees with previous *in vivo* data [5]. These results were likely due to the fact that the force equipollence was not considered in the formulation. With additional consideration of the force equipollence, the RFML formulation improved the performance of the knee model with ligaments.

Based on the knee model with ligaments, the RFML not only produced better estimates of the contact forces and the occurrence of the peak values, but also reduced the variability of the calculated forces for different objective functions (Figure 2). Apart from good estimates in the stance phase, the maximum contact forces with RFML were also less than 0.3 BW in magnitude during swing phase, which were more reasonable than those obtained with RM and RML which rose to a maximum of 1.0 BW. Contact forces measured using instrumented total knee replacements were less than about 0.3 BW during mid-swing phase of level walking [5,43]. The reduced variability in the calculated contact forces from different objective functions suggests that the calculated contact forces were not sensitive to the objective functions used as long as both moment and force equipollence were considered with a knee model.

Simultaneous consideration of moment and force equipollence on a knee model is also critical for estimating ligament forces. Excluding force equipollence from the problem formulation led to an imbalance of the forces at the joint with resultant force errors (Table 1), no matter whether the ligaments were included in the model (RM and RML). The unbalanced joint forces using RM (Table 1) should not be viewed as ligament forces as was often assumed in previous studies [5,16,24]. When only moment equipollence was considered (RML), the objectives were largely to minimize the sum of the muscle forces (or stresses) while counteracting the external moments. Since the lever arm lengths of the ligaments were much smaller than those of the muscles, the ligaments were required to transmit greater forces, leaving a maximum unbalanced force of about −4.4 BW (Table 1 and Figure 5).

The calculated ACL forces ranged from 1 BW to 3.7 BW, and those of the PCL from 3 BW to 7 BW (Figure 4), some of which were close to or much greater than the maximum strength of the ligaments reported in the literature (e.g., ACL: 2160 N [44], 638 N [45] and 633 N [46]; PCL: 1073 N [45] and 571 N [46]). These values could not be considered physiological during a non-strenuous activity such as level walking. With the additional inclusion of the force equipollence (RFML), the calculated ligament forces were quite different from those obtained using RML, both in magnitude and pattern, and appeared to be independent of the objective functions used (Figure 4). The ligament forces calculated using RFML were all less than 1.0 BW, and were within the maximum strength. The current results showed that when both moment and force equipollence were considered with a knee model, the selected objective functions showed obvious effects on the magnitudes and patterns of the predicted muscle forces, but less obviously on the calculated contact and ligament forces. A complete consideration of mechanical equipollence (i.e., RFML) with a knee model appeared to be more critical than the objective functions used for accurately estimating the ligament forces in the knee during walking.

In the current study, performance of different models and formulations was evaluated considering mechanical equipollence at the knee joint. Since recruitment and force production of some muscles of the knee, such as the hamstrings and gastrocnemius, may also be affected by the mechanical demands at the hip and ankle, further study considering mechanical equipollence at all three joints may be needed to confirm some of the current findings. Since direct measurement of the forces in the force-bearing structures was infeasible in living subjects, the current evaluation of the calculated contact forces had to be performed based on previous published data, including *in vivo* data from patients with instrumented total knee replacements. Therefore, a certain level of discrepancy between model predictions and experimental data should be expected, and the current results should be interpreted qualitatively.

The ligaments were assumed to be inextensible elements. If the ligaments were allowed to stretch under load, the femur would be displaced from its position as determined by the kinematic model of the knee and would translate on the tibial plateau until force and moment equilibrium were satisfied. Consider the ACL as an example; when the ACL was stretched to resist an anteriorly directed external force, the angle between its line of action and the tibial plateau would be reduced, producing a greater shear component. This in turn reduced the total force needed in the ACL for resisting this anteriorly directed shear force. Therefore, the assumption of inextensibility of the ligaments appeared to result in an overestimation of the total ligament forces, and the current results should be taken as an upper bound of the ligament forces. Further study would be needed to provide a more direct account of the effects of the inextensibility assumption.

The current study, performed on young healthy adults, aimed to investigate the influence of model complexity, problem formulation and optimization criteria on the prediction of the forces transmitted by the force-bearing structures in the knee. A similar study on pathological gait with muscle EMG data as a source of validation may be helpful for future clinical application of the techniques discussed in the current study.

## Conclusions

Model complexity and problem formulation affect the prediction of the forces transmitted by the force-bearing structures at the knee during normal level walking. Inclusion of the ligaments in a knee model enables the simultaneous consideration of equations of force and moment equipollence, which is required for accurately estimating the contact and ligament forces, and is more critical than the adopted optimization criteria.

## Abbreviations

ASIS: Anterior superior iliac spines; PSIS: Posterior superior iliac spines; ACL: Anterior cruciate ligament; PCL: Posterior cruciate ligament; MCL: Medial collateral ligament; LCL: Lateral collateral ligament; GRF: Ground reaction forces; GC: Gait cycle; BW: Body weight; PCSA: Physiological cross-sectional area; RMS: Root-mean-squared values; SNR: Signal-to-noise ratio; SQP: Sequential quadratic programming; RM: Problem formulation considering only moment equipollence in terms of force magnitudes of the muscles and articular contacts; RML: Problem formulation considering only moment equipollence in terms of force magnitudes of the ligaments, muscles and articular contacts; RFML: Problem formulation considering both force and moment equipollence in terms of force magnitudes of the ligaments, muscles and articular contacts; J1: Minimization of sum of muscle forces; J2: Minimization of sum of quadratic muscle forces; J3: Minimization of sum of cubic muscle forces; J4: Minimization of maximum of muscle forces; J5: Minimization of sum of muscle stresses; J6: Minimization of sum of quadratic muscle stresses; J7: Minimization of sum of cubic muscle stresses; J8: Minimization of the maximum of muscle stresses

## Competing interests

The authors declare that they have no competing interests.

## Authors’ contributions

All authors contributed to the conception and design of the study, the analysis and interpretation of data, and manuscript preparation. CCH contributed mainly to the analysis and interpretation of data, and manuscript preparation; TWL contributed to the conception of the study, experimental design, analysis and interpretation of data, and manuscript preparation; SCC carried out the experiments and data analysis. All authors read and approved the final manuscript.

## References

[B1] Zavatsky A O'Connor JJ A Model of human knee ligaments in the sagittal plane: part 1: response to passive flexion P I Mech Eng H 1992 206 3 125 134 10.1243/PIME_PROC_1992_206_280_02 1482508

[B2] Lu T O'Connor JJ Lines of action and moment arms of the major force-bearing structures crossing the human knee joint: comparison between theory and experiment J Anat 1996 189 Pt 3 575 585 8982833 PMC1167700

[B3] Taylor ME Tanner KE Freeman MR Yettram AL Stress and strain distribution within the intact femur: compression or bending? Med Eng Phys 1996 18 2 122 131 10.1016/1350-4533(95)00031-3 8673318

[B4] English TA Kilvington M *In vivo* records of hip loads using a femoral implant with telemetric output (a preliminary report) J Biomed Eng 1979 1 2 111 115 10.1016/0141-5425(79)90066-9 537339

[B5] Heinlein B Kutzner I Graichen F Bender A Rohlmann A Halder AM Beier A Bergmann G ESB Clinical Biomechanics Award 2008: Complete data of total knee replacement loading for level walking and stair climbing measured *in vivo* with a follow-up of 6–10 months Clin Biomech 2009 24 4 315 326 10.1016/j.clinbiomech.2009.01.011 19285767

[B6] Lu TW Taylor SJG O'Connor JJ Walker PS Influence of muscle activity on the forces in the femur: an *in vivo* study J Biomech 1997 30 11 1101 1106 10.1016/S0021-9290(97)00090-0 9456377

[B7] Collins JJ O'Connor JJ Muscle-ligament interactions at the knee during walking P I Mech Eng H 1991 205 1 11 18 10.1243/PIME_PROC_1991_205_256_02 1670070

[B8] Challis JH Kerwin DG An analytical examination of muscle force estimations using optimization techniques P I Mech Eng H 1993 207 3 139 148 10.1243/PIME_PROC_1993_207_286_02 8117365

[B9] Crowninshield RD Use of optimization techniques to predict muscle forces J Biomed Eng 1978 100 2 88 92

[B10] Seireg A Arvikar RJ The prediction of muscular load sharing and joint forces in the lower extremities during walking J Biomech 1975 8 2 89 102 10.1016/0021-9290(75)90089-5 1150683

[B11] Lu TW O'Connor JJ Taylor SJG Walker PS Validation of a lower limb model with *in vivo* femoral forces telemetered from two subjects J Biomech 1997 31 1 63 69 10.1016/S0021-9290(97)00102-4 9596539

[B12] Lu TW Lu CH Forces transmitted in the knee joint during stair ascent and descent J Mech 2006 22 4 289 297 10.1017/S1727719100000940

[B13] Lin HC Lu TW Hsu HC Comparisons of joint kinetics in the lower extremity between stair ascent and descent J Mech 2005 21 1 41 50 10.1017/S1727719100000538

[B14] Lu T Chien H Chen H Joint loading in the lower extremities during elliptical exercise Med Sci Sport Exer 2007 39 9 1651 1958 10.1249/mss.0b013e3180dc9970 17805099

[B15] Lu TW O'Connor JJ A three-dimensional computer graphics-based animated model of the human locomotor system with anatomical joint constraints J Biomech 1998 31 1 116(111)

[B16] Crowninshield RD Brand RA A physiologically based criterion of muscle force prediction in locomotion J Biomech 1981 14 11 793 801 10.1016/0021-9290(81)90035-X 7334039

[B17] Paul JP Kenedi RM Bioengineering studies of the forces transmitted by joints: II Engineering analysis Proceedings of Symposium on Biomechanics and Related Bio-engineering Topics: 1964; Glasgow 1964 Oxford: Pergamon Press 369 380

[B18] Morrison JB The mechanics of the knee joint in relation to normal walking J Biomech 1970 3 1 51 61 10.1016/0021-9290(70)90050-3 5521530

[B19] Davy DT Audu ML A dynamic optimization technique for predicting muscle forces in the swing phase of gait J Biomech 1987 20 2 187 201 10.1016/0021-9290(87)90310-1 3571299

[B20] Hardt DE Determining muscle forces in the leg during normal human walking—an application and evaluation of optimization methods J Biomed Eng 1978 100 2 72 78

[B21] Pedotti A Krishnan VV Stark L Optimization of muscle-force sequencing in human locomotion Math Biosci 1978 38 1 57 76 10.1016/0025-5564(78)90018-4

[B22] Patriarco AG Mann RW Simon SR Mansour JM An evaluation of the approaches of optimization models in the prediction of muscle forces during human gait J Biomech 1981 14 8 513 525 10.1016/0021-9290(81)90001-4 7276011

[B23] Seireg A Arvikar RJ A mathematical model for evaluation of forces in lower extremeties of the musculo-skeletal system J Biomech 1973 6 3 313 326 10.1016/0021-9290(73)90053-5 4706941

[B24] Herzog W Leonard T Validation of optimization models that estimate the forces exerted by synergistic muscles J Biomech 1991 24 1 31 39 1791180 10.1016/0021-9290(91)90375-w

[B25] Collins JJ The redundant nature of locomotor optimization laws J Biomech 1995 28 3 251 267 10.1016/0021-9290(94)00072-C 7730385

[B26] Cholewicki J Mcgill SM EMG assisted optimization: a hybrid approach for estimating muscle forces in an indeterminate biomechanical model J Biomech 1994 27 10 1287 1289 10.1016/0021-9290(94)90282-8 7962016

[B27] Gagnon D Larivière C Loisel P Comparative ability of EMG, optimization, and hybrid modelling approaches to predict trunk muscle forces and lumbar spine loading during dynamic sagittal plane lifting Clin Biomech 2001 16 5 359 372 10.1016/S0268-0033(01)00016-X 11390042

[B28] Vigouroux L Quaine F Labarre-Vila A Amarantini D Moutet F Using EMG data to constrain optimization procedure improves finger tendon tension estimations during static fingertip force production J Biomech 2007 40 13 2846 2856 10.1016/j.jbiomech.2007.03.010 17482624

[B29] Chen HL Lu TW Wang TM Huang SC Biomechanical strategies for successful obstacle crossing with the trailing limb in older adults with medial compartment knee osteoarthritis J Biomech 2008 41 4 753 761 10.1016/j.jbiomech.2007.11.017 18177877

[B30] Bisseling RW Hof AL Handling of impact forces in inverse dynamics J Biomech 2006 39 13 2438 2444 10.1016/j.jbiomech.2005.07.021 16209869

[B31] Wilson DR Feikes JD O'Connor JJ Ligaments and articular contact guide passive knee flexion J Biomech 1998 31 12 1127 1136 10.1016/S0021-9290(98)00119-5 9882045

[B32] O'Connor J Shercliff T Fitzpatrick D Biden E Goodfellow J Mechanics of the Knee 1990 New York: Raven

[B33] O'Connor JJ Can muscle co-contraction protect knee ligaments after injury or repair? J Bone Joint Surg Br 1993 75 1 41 48 8421032 10.1302/0301-620X.75B1.8421032

[B34] Meyer H Die mechanik des kniegelenkes Arch Anat Physiol Wiss Med 1853 497 547

[B35] Weber W Weber EF Mechanik der menschlichen gehwerkzeuge: Eine anatomisch-physiologische untersuchung 1836 Dietrich: Göttingen in der Dieterichschen buchhandlung

[B36] Brand RA Crowninshield RD Wittstock CE Pedersen DR Clark CR Van Krieken FM A model of lower extremity muscular anatomy J Biomed Eng 1982 104 4 304 310 10.1115/1.3138363 7154650

[B37] White SC Yack HJ Winter DA A three-dimensional musculoskeletal model for gait analysis. Anatomical variability estimates J Biomech 1989 22 8 885 893 10.1016/0021-9290(89)90072-9 2613724

[B38] Leardini A Cappozzo A Catani F Toksvig-Larsen S Petitto A Sforza V Cassanelli G Giannini S Validation of a functional method for the estimation of hip joint centre location J Biomech 1999 32 1 99 103 10.1016/S0021-9290(98)00148-1 10050957

[B39] Winter DA Biomechanics and motor control of human gait: normal, elderly and pathological Waterloo Biomechanics 1991 Canada: Waterloo Univ

[B40] Lu TW O'Connor JJ Bone position estimation from skin marker co-ordinates using global optimisation with joint constraints J Biomech 1999 32 2 129 134 10.1016/S0021-9290(98)00158-4 10052917

[B41] Wickiewicz TL Roy RR Powell PL Edgerton VR Muscle architecture of the human lower limb Clin Orthop Relat R 1983 179 275 283 6617027

[B42] Arora J Introduction to optimum design 2004 New York: McGraw-Hill

[B43] Kutzner I Heinlein B Graichen F Bender A Rohlmann A Halder A Beier A Bergmann G Loading of the knee joint during activities of daily living measured *in vivo* in five subjects J Biomech 2010 43 11 2164 2173 10.1016/j.jbiomech.2010.03.046 20537336

[B44] Woo SLY Hollis JM Adams DJ Lyon RM Takai S Tensile properties of the human femur-anterior cruciate ligament-tibia complex The effects of specimen age and orientation Am J Sports Med 1991 19 3 217 225 10.1177/036354659101900303 1867330

[B45] Kennedy JC Hawkins RJ Willis RB Danylchuk KD Tension studies of human knee ligaments J Bone Joint Surg A 1976 58 3 350 355 1262366

[B46] Trent PS Walker PS Wolf B Ligament length patterns, strength, and rotational axes of the knee joint Clin Orthop Relat R 1976 117 6 263 270 1277674

